# Supplementary figures and images for: Molecular Genetic Characteristics of Different Scenarios of Xylogenesis on the Example of Two Forms of Silver Birch Differing in the Ratio of Structural Elements in the Xylem
Source: Plants (Basel). 2021 Aug 2;10(8):1593. doi: 10.3390/plants10081593 (PMC8400816; doi:10.3390/plants10081593)

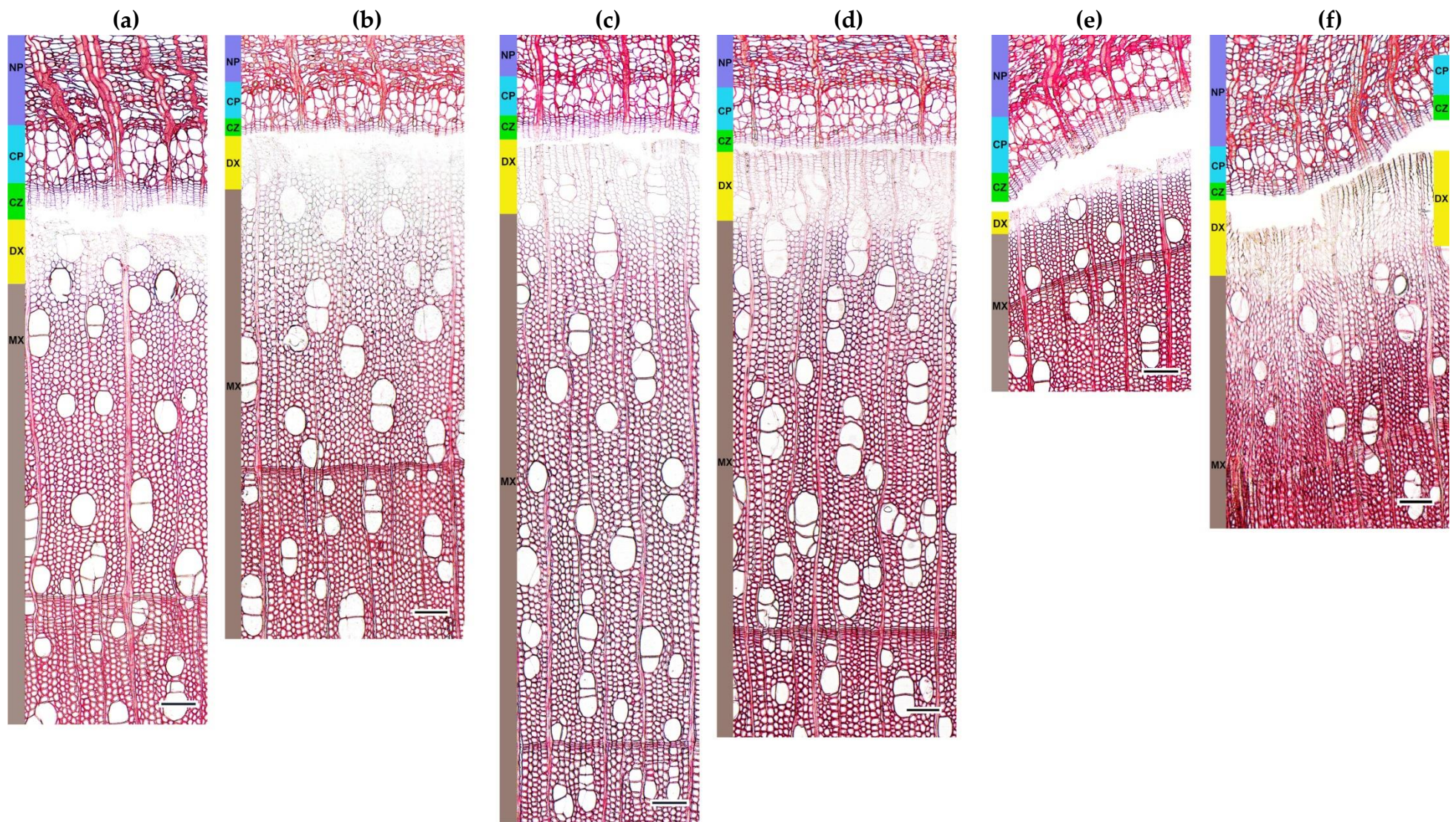

Supplementary Materials: Figure S1

Supplement: Supplementary file 1 [file plants-10-01593-s001.zip › Supplementary Figure S1.pdf]
